# Distinct Spatial Patterns of SAR11, SAR86, and Actinobacteria Diversity along a Transect in the Ultra-oligotrophic South Pacific Ocean

**DOI:** 10.3389/fmicb.2016.00234

**Published:** 2016-03-08

**Authors:** Nyree J. West, Cécile Lepère, Carmem-Lara de O. Manes, Philippe Catala, David J. Scanlan, Philippe Lebaron

**Affiliations:** ^1^Sorbonne Universités, UPMC Univ Paris 06, CNRS, Observatoire Océanologique de Banyuls, Banyuls-sur-Mer, France; ^2^Université Clermont Auvergne, Université Blaise Pascal, CNRS, Laboratoire Microorganismes Génome et Environnement, Aubière, France; ^3^Sorbonne Universités, UPMC Univ Paris 06, CNRS, Laboratoire de Biodiversité et Biotechnologies Microbiennes, Observatoire Océanologique, Banyuls-sur-Mer, France; ^4^Sorbonne Universités, UPMC Univ Paris 06, CNRS, Laboratoire d’Océanographie Microbienne, Observatoire Océanologique, Banyuls-sur-Mer, France; ^5^School of Life Sciences, University of Warwick, Coventry, UK

**Keywords:** Actinobacteria, SAR11, SAR86, 16S rRNA, SSCP, BIOSOPE, South Pacific Gyre

## Abstract

Distinct distribution patterns of members of the major bacterial clades SAR11, SAR86, and Actinobacteria were observed across a transect from the Marquesas islands through the ultra-oligotrophic South Pacific Gyre into the Chilean upwelling using 16S rRNA gene sequencing and RNA–DNA fingerprinting. Three different Actinobacteria sequence clusters belonging to “*Candidatus* Actinomarinidae” were localized in the western half of the transect, one was limited to the gyre deep chlorophyll maximum (DCM) and sequences affiliated to the OCS155 clade were unique to the upwelling. The structure of the surface bacterial community was highly correlated with water mass and remained similar across the whole central gyre (1300 nautical miles). The surface hyperoligotrophic gyre was dominated (>70% of all sequences) by highly diverse SAR11 and SAR86 operational taxonomic units and these communities were significantly different from those in the DCM. Analysis of 16S rRNA fingerprints generated from RNA allowed insights into the potential activity of assigned bacterial groups. SAR11 and *Prochlorococcus* showed the highest potential activity in all water masses except for the upwelling, accounting together for 65% of the total bacterial 16S rRNA in the gyre surface waters in equal proportions whereas the contribution of SAR11 decreased significantly at the DCM.

## Introduction

The oligotrophic regions of the world’s oceans are inhabited primarily by small microorganisms (<3 μm), the picophytoplankton and heterotrophic bacteria that are adapted for survival under nutrient limiting conditions. Despite their low productivity, these regions, by their sheer size, have an important influence on global biogeochemical cycles (Karl et al., 1996). The South Pacific Gyre (SPG) is the largest oligotrophic region (Polovina et al., 2008) yet remains under-sampled compared to the North Pacific Gyre, and the North Atlantic Gyre, the sites of the oceanographic stations HOT (Hawaiian Ocean Time series) and BATS (Bermuda Atlantic Time Series). These stations have been regularly sampled for over two decades providing a wealth of physicochemical and biological data. Seasonal water column mixing is an annual feature at BATS, and consequently, changes in the bacterial community structure are more marked at BATS than at HOT (Morris et al., 2005; Carlson et al., 2009; Treusch et al., 2009; Giovannoni and Vergin, 2012). The alphaproteobacterial clade SAR11 is ubiquitous in coastal and open ocean environments (Morris et al., 2002), and dominates the heterotrophic bacterial fraction at HOT (Eiler et al., 2009), BATS (Carlson et al., 2009), and also in the South Atlantic Gyre (Morris et al., 2012). SAR11 exhibits high microdiversity (Brown and Fuhrman, 2005; Vergin et al., 2013) and has been divided into 5–9 subclades according to 16S rRNA gene sequences (Suzuki et al., 2001; Morris et al., 2005; Vergin et al., 2013) or into more than 10 subclades based on 16S–23S rRNA ITS comparisons (Fuhrman and Steele, 2008; Brown et al., 2012). The different subclades represent distinct ecotypes whose dynamics can be influenced by temperature, depth, seasonal overturn or by phytoplankton abundance (Brown et al., 2005, 2012; Carlson et al., 2009; Eiler et al., 2009; Vergin et al., 2013; Salter et al., 2015). Other oligotrophic bacterial clades that coexist with SAR11 include the alphaproteobacterial clades SAR116 and OCS116, and the gammaproteobacterial clade SAR86. The SAR86 group is also divided into several subclades that probably represent different ecotypes (Suzuki et al., 2001; Treusch et al., 2009; Dupont et al., 2012). SAR86 and SAR116 exhibit similar seasonal dynamics at BATS, increasing in relative abundance in the oligotrophic euphotic zone with the onset of summer stratification (Morris et al., 2005; Treusch et al., 2009). In contrast, OCS116, SAR11 and marine Actinobacteria abundance increases with the spring phytoplankton bloom at BATS (Morris et al., 2005), but there is also a continuous OCS116 population in the deep chlorophyll maximum (DCM) throughout the year (Treusch et al., 2009).

In contrast to the previously described groups whose spatiotemporal patterns can be resolved at the sub-clade or ecotype level, marine Actinobacteria are a relatively poorly studied group, despite their wide oceanic distribution (Rusch et al., 2007), high diversity (Jensen and Lauro, 2008), and instances of high relative abundance (Morris et al., 2005, 2010). Nonetheless, two marine Actinobacteria operational taxonomic units (OTUs) with distinct temperate or tropical distributions were identified in the Global Ocean Sampling expedition dataset (Rusch et al., 2007) and ARISA defined 16S–23S rRNA ITS Actinobacteria OTUs have been correlated with spring phytoplankton blooms (Brown et al., 2005). Community network analysis of ARISA OTUs has also revealed that different Actinobacteria OTUs are significantly correlated with different abiotic and biotic factors and that they also show different temporal patterns (Fuhrman and Steele, 2008; Needham et al., 2013).

Pelagic heterotrophic bacteria obtain a significant fraction of their carbon demand from phytoplankton-derived organic matter. Indeed in the SPG, there appeared to be a strong coupling between primary production and bacterial production (Van Wambeke et al., 2008b). In these hyperoligotrophic regions where phytoplankton biomass is exceedingly low, little is known of how phytoplankton species composition could influence the heterotrophic bacteria community structure. The major primary producer in these regions is usually the photosynthetic prokaryote *Prochlorococcus* but photosynthetic picoeukaryotes can also contribute significantly to total biomass (Grob et al., 2007).

Very few oceanographic cruises have passed through the SPG and there is only patchy information on the structure of heterotrophic bacterial communities in this region. One paper presented microbial composition data from a transect from one station in the SPG toward the rim of the gyre (Yin et al., 2013) and a recent study focused on the archaeal and bacterial populations in the DCM of several gyre stations (Walsh et al., 2015). Samples from the BIOSOPE oceanographic cruise taken along a transect of around 8000 km in the South East Pacific Ocean presented an ideal opportunity to study patterns of microbial diversity in the context of a suite of environmental variables and different phytoplankton distributions (Claustre et al., 2008). The goals of this study were to (i) characterize bacterial diversity in the unique hyperoligotrophic habitat of the SPG, (ii) to determine patterns of bacterial diversity distribution across the South Pacific Ocean and gain insights into potential activity of the bacterial community through a RNA–DNA fingerprinting approach, and (iii) to determine the influence of biotic and abiotic variables on changes in bacterial community structure.

## Materials and Methods

### Study Sites

**FIGURE 1:**
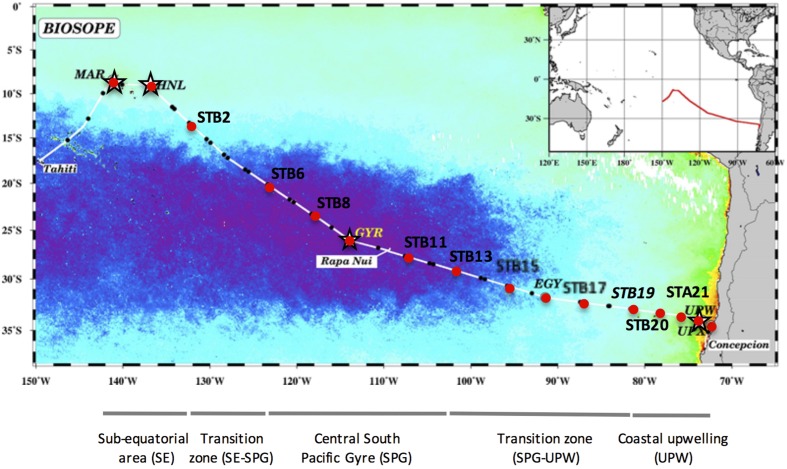
**Map of stations sampled during the BIOSOPE cruise, superimposed on a SeaWiFS composite image of Chl*a* concentration in the upper layer for November–December 2004.** The purple area indicates the extent of the hyperoligotrophic gyre. The stations analyzed by SSCP are marked with a red dot and those used for 16S rRNA gene clone library construction are indicated with a star. The different water masses illustrated under the map are those defined previously (Claustre et al., 2008).

The BIOSOPE (BIogeochemistry and Optics South Pacific Experiment) cruise track crossed the South Pacific Ocean from the west of the Marquesas Archipelago to the coastal waters of Chile (Claustre et al., 2008) during the Austral summer between the 26 October and the 11 December 2004. The stations sampled are indicated in **Figure 1** and consisted of six long-term (>2 days) stations (MAR, HNL, GYR, EGY, UPW, and UPX) and 21 short-term (<5 h) stations. The samples analyzed in this study are indicated by a black dot and 16S rRNA gene clone libraries prepared at stations MAR, HNL, GYR and UPW, are marked with a star (**Figure 1**).

### Sample Collection

Seawater samples were collected at multiple depths at the long-term stations and at the surface and DCM depths at the short-term stations using a CTD-rosette system equipped with 21 L × 12 L Niskin bottles. The water samples were successively filtered through 0.8 μm pore-size filters and then onto 0.2 μm pore-size Sterivex cartridges (Millipore, Billerica, NY, USA) or onto 47 mm, 0.2 μm pore-size Nuclepore polycarbonate filters (Whatman, Maidstone, UK) except for stations MAR and STB13 (successive filtration through 3.0 μm pore-size filters and then onto 0.2 μm Sterivex). Filtered volumes were noted and ranged between 4.5 and 8.1 L for the Sterivex collections and between 0.75 and 1.0 L for the polycarbonate filter collections. The filters and Sterivex cartridges were immediately stored in liquid nitrogen then at –80°C until nucleic acid extraction. DNA extractions were carried out on Sterivex cartridges from all stations except for HNL, STB8, STB11, and STB19 where only polycarbonate filters were available.

### Environmental and Biological Variables

Environmental data for pigments (Ras et al., 2008), inorganic nutrients (Raimbault et al., 2008), picoplankton abundance (Grob et al., 2007) and bacterial production (Van Wambeke et al., 2008a) was obtained from http://www.obs-vlfr.fr/proof/vt/op/ec/biosope/bio.htm.

### DNA Extraction

DNA and RNA were simultaneously extracted with the AllPrep kit (Qiagen, Chattsworth, CA, USA) essentially as described previously (Manes et al., 2010). 425 μl TES lysis buffer (50 mM Tris, 40 mM EDTA, 0.75 M sucrose, prepared with DEPC treated water) was added to the sterivex units (with outlet capped). The sterivex were closed with parafilm and placed in hermetically sealed bags before carrying out three freeze/thaw (liquid N2/water bath at 65°C) cycles. Lysozyme treatment was performed by adding 25 μl freshly prepared lysozyme solution (36 mg ml^-1^ in lysis buffer) and incubating at 37°C for 45 min on a rotary shaker. Cell lysis was completed by adding 8 μl Proteinase K solution (20 mg ml^-1^ in lysis buffer, 0.2 mg ml^-1^ final) and 26 μl of 20% v/v SDS (1% final), inverting gently and incubating at 55°C with gentle agitation for at least 1–2 h. β-mercaptoethanol was added to the RLT Plus buffer according to the manufacturer’s instructions and then 1550 μl of this buffer was added to each sterivex. Sterivex were incubated 20 min on a rotary shaker and then the lysates were recovered into microtubes. Lysate processing and further purification of the DNA and RNA on the specific columns were carried out according to the manufacturer’s instructions. Extraction of nucleic acids from polycarbonate filters was carried out by cutting the filters into small pieces with sterile scissors, placing the pieces in a 2 ml microtube and then carrying out the same protocol as for the sterivex. The quality of the DNA and RNA was verified by agarose gel electrophoresis and DNA concentrations were measured by the Quant-it Picogreen assay (Invitrogen, Carlsbad, CA, USA). cDNA was synthesized immediately from extracted RNA using MMLV reverse transcriptase (Promega) according to manufacturer’s instructions. Briefly, 13 μl RNA was mixed with 1 μl of the 16S rRNA gene specific primer w34-rev (5^′^-TTACCGCGGCTGCTGGCAC-3^′^; 10 μM; Lee et al., 1996) and denatured at 70°C for 5 min before cooling on ice. 11 μl of MMLV mix (5 μl dNTPs, 5 μl MMLV buffer, and 1 μl MMLV reverse transcriptase) was added and cDNA synthesis carried out for 1 h at 42°C. MMLV was inactivated by incubation at 94°C for 15 min. DNA, RNA, and cDNA samples were stored at –80°C until use.

### Construction of 16S rRNA Gene Clone Libraries

16S rRNA gene clone libraries were constructed for samples collected from the surface and DCM depths from stations MAR (15 and 40 m), HNL (5 and 80 m), GYR (5 and 180 m), and UPW (5 and 35 m). The method was based on that described previously (West et al., 2008) using the bacteria specific primers 27F_MOD_ (5^′^-AGRGTTTGATCMTGGCTCAG-3^′^) and 1492R_MOD_ (5^′^-TACGGYTACCTTGTTAYGACTT-3^′^; Vergin et al., 1998). Ten replicate PCR reactions were performed to reduce PCR bias. Reactions included a mixture of two DNA polymerases [1 U SuperTaq Polymerase (HT Biotechnology, Cambridge, UK), and 0.05 μl Taq Advantage (Clontech)], 0.2 μM primers, 0.2 mM each dNTP, 5 μg BSA, 1X SuperTaq PCR buffer, 2.5 mM Mg^2+^ and 15 ng DNA in a total reaction volume of 25 μl. Cycling conditions were 3 min at 94°C followed by 20 cycles of 1 min at 94°C, 1 min at 50°C and 2 min at 72°C with a final extension of 10 min at 72°C. The presence of bands of the correct size was verified by agarose gel electrophoresis. Reconditioning PCR to reduce heteroduplexes (Thompson et al., 2002) involved pooling replicate PCR reactions, purifying the products with the Qiaquick PCR Purification Kit (Qiagen), mixing 10 μl of pooled products mixed with 90 μl of fresh PCR mix (as above) and cycling a further three times with the above PCR conditions. PCR products (2 μl) were cloned immediately with the TOPO TA^®^ cloning kit pCR2.1 (Invitrogen) according to the manufacturer’s instructions.

### Sequencing and Phylogenetic Analysis

Plasmid DNA (192 clones for each library) was sequenced using the BigDye^TM^ terminator kit and a 3730xl Automatic Sequencer (Applied Biosystems, Foster City, CA, USA) by Macrogen (Seoul, South Korea) using primers 27F (5^′^-AGAGTTTGATCMTGGCTCAG-3^′^) and 1492R (5^′^-TACGGYTACCTTGTTACGACTT-3^′^). Sequences were trimmed for quality and length (>500 bp) using the CodonCode Aligner software (CodonCode Corporation, Dedham, MA, USA). Alignment of the sequences was achieved with the mothur program (Schloss et al., 2009) using the SILVA seed alignment. Non-aligning sequences were removed from the dataset. The alignment was imported into the non-redundant SILVA 104 ARB database (http://www.arb-silva.de/). The sequence alignment was further improved in ARB by using secondary structure information. Sequences were then exported phylum by phylum together with a reference sequence for chimera screening by the Mallard program (Ashelford et al., 2006). Potential chimeras were then rechecked individually with Pintail (Ashelford et al., 2005). Chimeras were also screened with the UCHIME algorithm (Edgar et al., 2011; options -minh 0.8, -noskipgaps -noskipgaps 2, -abskew = 1) after clustering with USEARCH and UCLUST (Edgar, 2010) and the results checked manually.

The improved ARB alignment (with chimeras removed) was exported and sequences were then analyzed with the mothur program. The initial alignment of 1353 sequences was filtered to optimize the maximum length of sequence (645 positions retained) whilst retaining the maximum number of sequences. The 1275 sequences remaining after filtering were clustered into OTUs either at 97 or 99% similarity with the average neighbor method and OTUs were classified using the SILVA_119_SSURef_Nr99_database. It has been shown that clustering at 99% similarity can reveal the natural grouping of marine bacterioplankton into microdiverse clusters with similar ecological roles that would otherwise be missed at a 97% cut-off (Acinas et al., 2004; Malmstrom et al., 2007; West et al., 2008). For this reason, all further sequence analyses were performed with the 99% sequence similarity cut-off.

The phylogenetic tree of Actinobacteria sequences was constructed from almost full-length 16S rRNA gene sequences using a filtered alignment (Filter by base frequency; 50% minimal similarity) from ARB, resulting in 1100 positions to only allow comparisons between unambiguously aligned positions. These filtered alignments were used for the construction of a tree using MrBayes v. 3.2 (Altekar et al., 2004).

Bacterial 16S rRNA gene sequences were submitted to GenBank under the accession numbers KM222828-KM224122. The *Prochlorococcus* 16S rRNA sequences are already published under the accession numbers HQ232982-HQ233045 (West et al., 2011).

### Single Strand Conformation Polymorphism (SSCP) Analysis

Synthesis of cDNA from RNA and subsequent SSCP analysis of the PCR-amplified DNA and cDNA was carried out as described previously (West et al., 2008).

Short fragments (∼200 bp) of the V3 region of the 16S rRNA gene were amplified from DNA or from cDNA using the bacterial specific primers w49dir (5^′^-ACGGTCCAGACTCCTACGGG-3^′^; Delbès et al., 1998) and w34rev (5^′^-TTA CCG CGG CTG CTG GCA C-3^′^). Primer w34rev was 5^′^-labeled with the fluorochrome 5^′^-tetrachloro-fluorescein phosphoramidite (TET). PCR reactions (50 μl) contained 1 μl of diluted DNA (0.2 ng/μl) or cDNA (0.1 ng/μl), 0.3 μM primers, 0.2 mM each dNTP, 1–1.5 U of *pfu* DNA polymerase and 1X *pfu* buffer (Promega). The reactions were cycled using an initial denaturation of 1 min at 94°C followed by 25 cycles of 30 s at 94°C, 30 s at 61°C and 30 s at 72°C with a final extension of 10 min at 72°C. Amplification products were verified by agarose gel electrophoresis and their concentrations estimated by comparison with molecular size markers (Smart Ladder, Promega). Dilutions were made (2–40 fold) in molecular grade water (Sigma) and 1 μl of each dilution was mixed with 18.8 μl deionised formamide (Hi-Di formamide^TM^; Applied Biosystems) and 0.2 μl of the internal size standard GeneScan-400HD (Rox; Applied Biosystems). Samples were denatured at 94°C for 5 min and then placed immediately in a water/ice bath for 10 min. Fragments were separated by capillary electrophoresis SSCP (CE-SSCP) as described previously (Delbès et al., 2000) using a ABI310 Genetic Analyser (Applied Biosystems) with electrophoresis at 12 kV and 30°C for 30 min per sample. The electrophoretograms were analyzed by the Genescan software (Applied Biosystems) using the second-order least square size calling method to normalize mobilities between different runs. Peak relative size, height and area data were exported for each sample and the peaks binned manually for the whole dataset. Relative peak abundance for the 35 binned peaks for a given SSCP profile was calculated as a percentage of total area under each corresponding profile. These relative abundance data were than analyzed (below) by the PRIMER-E software (Plymouth Marine Laboratory, UK). Peak assignment was done as described previously by analyzing selected clones by SSCP and superimposing the clones’ profiles on the stations’ profiles (West et al., 2008). One or two clones were selected for the OTUs comprising four or more sequences except for the SAR11 and SAR86 clades where the number of OTUs was higher. For these clades, clones from 9 to 4 OTUs respectively were analyzed (the most abundant).

### Statistical Testing

Statistical analysis was performed using the PRIMER-E software (PRIMER-E Ltd, UK) and the R software environment (R Core Team, 2015). SSCP relative abundance peak data was transformed with the square root transformation and similarity matrices created using the Bray–Curtis algorithm. Clustering was done using the group average. The ANOSIM (analysis of similarities) routine was performed to test the null hypothesis that there were no differences of bacterial diversity either between the surface or DCM samples, between different trophic regimes [surface mixed layer (SML) depths only] or between different water masses as defined previously (Claustre et al., 2008; SML depths only).

CCA ordinations were carried out for stations MAR-STB19 using the vegan package in R. The upwelling stations were considered as outliers since they showed extreme values of nutrients and chlorophyll and were thus excluded from the analysis. Ordinations were tested using different combinations of abiotic and biotic variables. Abiotic variables included temperature (Temp), salinity (Sal), depth (DCM) and the nutrients NO_3_, NO_2_, NH_4_, PO_4_. Biotic variables included total chlorophyll *a* (Tchl*a*) and picophytoplankton cell abundances [*Prochlorococcus* (Pro), *Synechococcus* (Syn), picoeukarytoes (PEuk)]. To reduce the skewness of the variables, a normalizing transformation was carried out. All abiotic variables except for Temp and Sal were log transformed and biotic variables were square root transformed. Collinear variables were removed and selection of the most significant explanatory variables was guided by automatic model building with the vegan package followed by manual model building. The significance of the CCA axes and the significance of the individual variables used in the models were tested by permutation (1000 permutations). Even though only two variables were significant at any one time, several combinations of significant variables were possible and those with a variance inflation factor (VIF) of less than ten are shown in the ordination plots.

To explore the correlation between bacterial assemblage composition and a range of environmental variables or between variables associated specifically to phytoplankton, Mantel tests (Mantel, 1967) were done on the bacterial SSCP data reported here, from surface and DCM depths, or from all depths treated together. The environmental variables used in the analysis were Sal, Temp, Chl*a*, dissolved oxygen, nutrients NO_3_, NO_2_, NH_4_, PO_4_, *Synechococcus, Prochlorococcus* and picoeukaryote abundances. Pigment data included chlorophyll *a*, *b*, *c1*, *c2*, peridinin, 19^′^-butanoyloxyfucoxanthin, fucoxanthin, prasinoxanthin, 19^′^-hexanoyloxyfucoxanthin, alloxanthin, lutein, neoxanthin, violaxanthin, diadinoxanthin, zeaxanthin, and phaeophytin *a*. Correlations were carried out between a Bray–Curtis community dissimilarity matrix (SSCP diversity data) and a matrix of environmental Euclidian distances. The statistic was tested for significance (α = 0.05) using 999 permutations.

## Results

### Bacterial Community Structure in Surface Waters and the DCM along the BIOSOPE Transect

**FIGURE 2:**
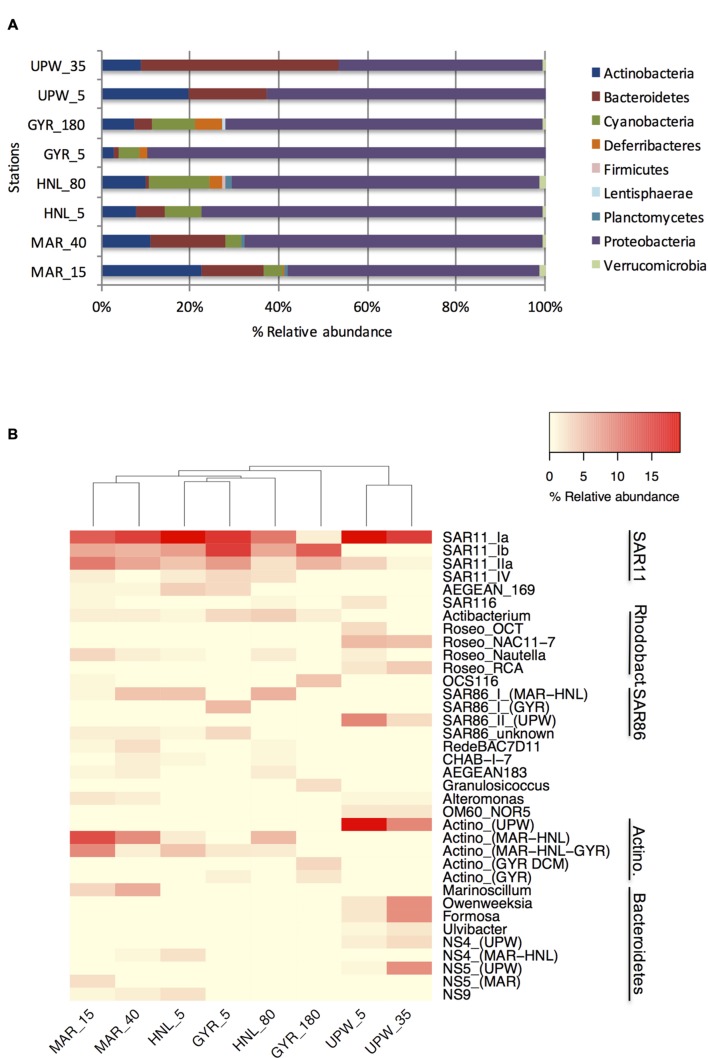
**Phylogenetic composition of the 16S rRNA gene clone library sequences for the surface and DCM depths of the four long stations shown at the phylum level **(A)** or at the OTU/clade level **(B)**.** Sample depths are indicated after the station name. For clarity, the relative abundance of those multiple OTUs with the same taxonomic classification (notably for SAR11, SAR86, and Actinobacteria) were grouped together according to phylogenetically established clades.

Clone libraries of 16S rRNA genes were prepared from surface and DCM depths at four stations along the BIOSOPE transect (see **Figure 1**) to determine if there were differences in bacterial diversity between the chlorophyll enriched depths and the surface waters, as well as across the various trophic regimes encountered along the transect. Even at the phylum level, there were important differences in the dominant phylogenetic groups between the surface GYR station and the other surface stations, notably the very low number of Bacteroidetes and Actinobacteria sequences, and the dominance of Proteobacteria sequences (**Figure 2A**). The heatmap in **Figure 2B** illustrates at a finer taxonomic level, the differential distributions of the most abundant OTUs (Supplementary Table S2), presented either as individual OTUs or grouped into taxonomically recognized clades (SAR11, SAR86, Actinobacteria). The phylogenetic similarity of the microbial community structure in each sample was also estimated using the UniFrac metric (Lozupone and Knight, 2005) and the result is shown as a cluster dendrogram above the heatmap (**Figure 2B**). The surface and DCM depth communities were similar to each other for the stations MAR and UPW as observed in the heatmap and the associated UniFrac cluster dendrogram. This is supported by the UniFrac significance test that gave non-significant *p*-values for the MAR (*p* = 0.28) and UPW (*p* = 0.06) stations. This is in contrast to the HNL and GYR stations where significant (*p* = 0.01) and highly significant (*p* = <0.001) differences respectively, were found between the surface and DCM libraries. Alpha diversity was always higher in the DCM library compared to the surface (**Table 1**; see also Supplementary Figure S1).

**Table 1:** Alpha diversity for the surface and DCM libraries from the four stations MAR, HNL, GYR, and UPW normalized to 140 sequences for each library with sequence clustering into OTUs at 99% similarity.

Station	N° clones	Coverage	N° OTUs	InvSimpson (1/D)	Simpson evenness	Shannon	Diversity rank
MAR_15	172	0.62(0.81)	74(41)	39.55(12.21)	0.53(0.30)	3.90(2.95)	6
MAR_40	165	0.56(0.85)	84(42)	52.88(12.36)	0.63(0.29)	4.11(3.03)	4
HNL_5	141	0.54(0.84)	86(40)	59.69(10.58)	0.69(0.26)	4.16(2.94)	3
HNL_80	140	0.53(0.87)	91(41)	98.28(15.35)	1.08(0.37)	4.32(3.13)	1
GYR_5	177	0.62(0.82)	74(40)	45.05(10.21)	0.61(0.26)	3.95(2.86)	5
GYR_180	165	0.46(0.74)	94(57)	74.85(18.19)	0.80(0.32)	4.28(3.46)	2
UPW_5	158	0.76(0.79)	52(43)	12.99(9.45)	0.25(0.22)	3.18(2.87)	8
UPW_35	157	0.75(0.86)	55(39)	23.00(17.19)	0.42(0.44)	3.47(3.13)	7


*Values at the 97% similarity cutoff are shown in brackets*.

Although, the taxonomic compositions of the HNL and GYR DCM samples appeared similar at the phylum level, there were only seven shared major OTUs, most of which were affiliated to SAR11. These significant phylogenetic differences were supported by UniFrac analysis (*p* < 0.001). In the surface waters of the GYR station, the Proteobacteria sequences accounted for a staggering 90% of all sequences and were comprised mainly of SAR11 and SAR86 OTUs representing 53 and 17% of the total sequences respectively. SAR11 sequences were also represented by the highest number of OTUs (45) in stark contrast to the surface upwelling station where the number was significantly lower (11 OTUs). The GYR DCM showed a lower relative abundance of Proteobacteria sequences (70%) and increases in abundance of the phyla Actinobacteria, Bacteroidetes, Deferribacteres, and Cyanobacteria (*Prochlorococcus*). Interestingly several SAR11, SAR86, and OCS116 OTUs were either more abundant in the GYR DCM than at the other stations or appeared unique to this sample. The bacterial phylogenetic diversity in the upwelling was dramatically different from the mesotrophic stations MAR and HNL and from the hyperoligotrophic gyre (**Figure 2B**; Supplementary Figure S2). These bacterial communities were dominated by SAR11 S1a, several *Roseobacter* OTUs, a single SAR86 OTU, a single Actinobacteria OTU and several OTUs attributed to Bacteroidetes. With the exception of the SAR11 S1a OTU and SAR11 IIa OTUs, the vast majority of the other OTUs were specific to the UPW station. Reduced microdiversity of the major SAR11 and SAR86 clades was observed at UPW with the majority of SAR11 sequences (>60%) falling into a single OTU and the presence of a single major SAR86 OTU corresponding to clade II (Suzuki et al., 2004) that was unique to UPW. Distinct distribution patterns across the transect were visible for the majority of the dominant OTUs presented (**Figure 2B**) notably for the SAR11, *Roseobacter* and SAR86 clades, and for different *Actinobacteria* and Bacteroidetes OTUs.

**FIGURE 3:**
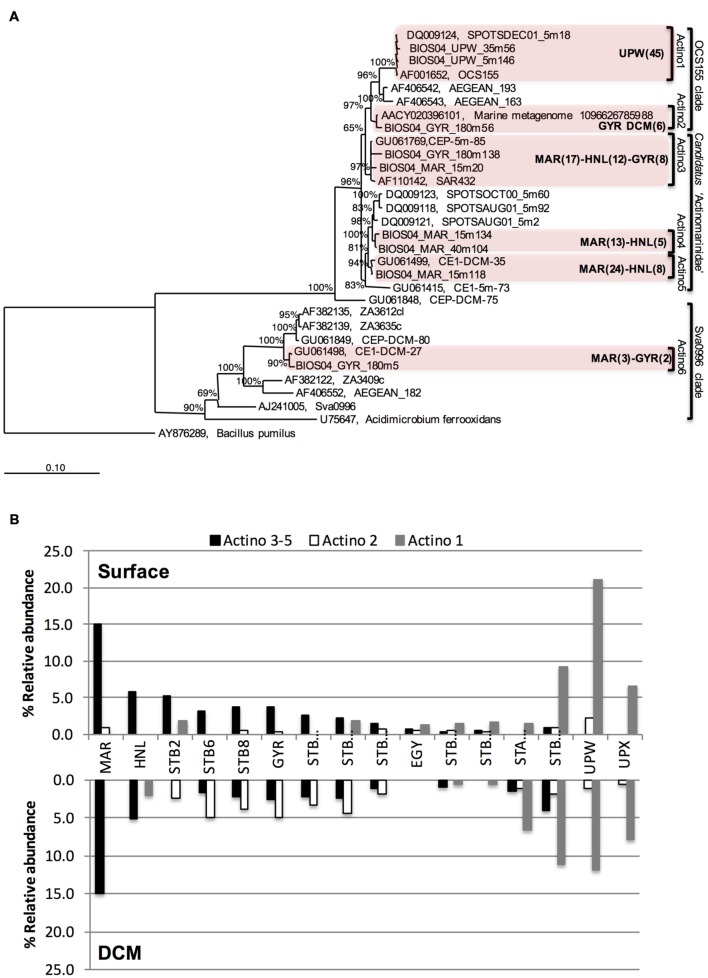
**(A)** Bayesian-inferred phylogenetic tree of almost full-length Actinobacteria sequences showing the clustering into six different clades depending on their geographical origin. The number of sequences (partial and full-length) attributed to each clade is presented in brackets for a given station. **(B)** Differential distribution of three assigned Actinobacteria OTUs dominating at MAR and HNL and GYR surface (Actino 3–5), at the GYR DCM (Actino 2), or at UPW (Actino 1) determined from the relative abundance of SSCP peaks from DNA at surface or DCM depths.

To further explore the phylogenetic relationships between the different Actinobacteria OTUs, a Bayesian tree was constructed from almost full length 16S rRNA gene sequences (**Figure 3A**). Actinobacteria sequences were affiliated to three different clades: (i) the OCS155/OM1 clade comprising two clusters, one unique to the UPW (Actino 1) and the other one localized at GYR DCM (Actino 2); (ii) the newly proposed sub-class of Actinobacteria known as “*Candidatus* Actinomarinidae” comprising two closely related Actinobacteria clades (Actino 4 and 5) that were only recovered at the MAR and HNL stations and a third cluster of sequences recovered from MAR, HNL, and GYR (Actino 3); and (iii) the Sva0996 clade (Actino 6) represented by a few clones. Although, the Actino 3 group clustered apart from Actino 4 and Actino 5, a PROBE MATCH search with the ARB software and the “*Candidatus* Actinomarinidae” probe suggested its affiliation to this group. The differential distribution of the Actino 1 UPW clade, the Actino 2 GYR DCM clade and the Actino 3, 4, and 5 clades present at MAR, HNL, and GYR observed in the clone libraries was also confirmed across the whole transect by SSCP analysis (**Figure 3B**). The higher abundance of the Actino 2 OTU in the DCM of the gyre was also consistent with the clone libraries.

### Bacterioplankton Diversity and Activity across the BIOSOPE Transect

Bacterial diversity and activity at multiple stations across the transect (**Figure 1**) were assessed by SSCP analysis of a 16S rRNA gene fragment amplified from extracted DNA to indicate the presence of bacterial groups or from synthesized cDNA from co-extracted RNA to reveal the potentially active bacterial groups. Note that when we refer to activity and presence, this refers to the relative changes of one OTU to another in the same sample but does not indicate absolute changes in cell abundance or activity *in situ*. Furthermore, the relative activity from the RNA profiles reflects the potential activity of the assigned OTUs or their protein synthesizing capacity (Blazewicz et al., 2013).

**FIGURE 4:**
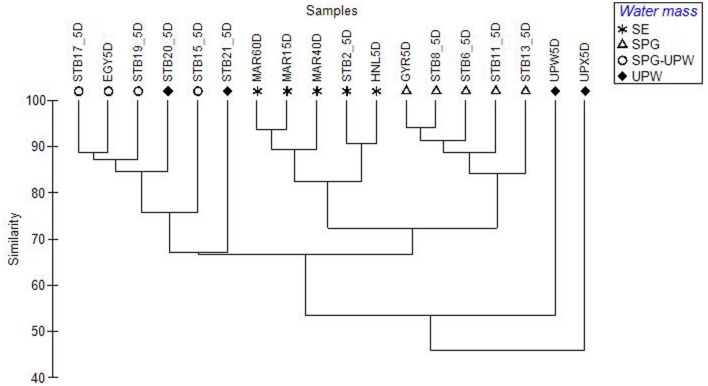
**Dendrogram constructed from a Bray–Curtis dissimilarity matrix of SSCP profiles from surface mixed layer samples across the BIOSOPE transect, showing the structuring of the bacterial communities according to water mass**.

Surface DNA samples from the hyperoligotrophic central gyre from stations STB6-STB13 clustered tightly together (**Figure 4**; Supplementary Figure S2) showing a high similarity of bacterial diversity over a distance of 1300 nautical miles. The corresponding DCM samples clustered separately at >80% similarity and formed a larger cluster with the DCM samples from the eastern gyre (STB15-STB19; Supplementary Figure S2). The bacterial diversity at the coastal upwelling was very different between the stations UPW and UPX (<60% similarity in their SSCP profiles) despite being only 60 nautical miles apart, and these stations also clustered apart from all the other stations (**Figure 4**). To determine the most significant factors explaining the dissimilarity of the bacterial diversity between the samples along the transect, based on either the depths sampled (surface and DCM), the trophic regime (chl*a* content) or on water mass, one-way ANOSIM tests were performed. The most significant differences between groups were found between the different water masses (Global *R* = 0.701, *p* = 0.001) with additional significant pairwise differences between the SE, SPG and SPG-UPW water masses (*R* > 0.9, *p* = 0.008). Differences between samples based on trophic regime and depth were less significant (Global *R* = 0.411, *p* = 0.003 and Global *R* = 0.183, *p* = 0.001 respectively) although highly significant differences between surface and DCM samples were observed for the SPG stations (Global *R* = 1, *p* = 0.008%).

Assignment of a limited number of peaks was achieved as described previously (West et al., 2008) by SSCP analysis of clones from the most abundant OTUs and the subsequent construction of a database of peak migration. Three different peaks could be assigned to (i) the Actinobacteria clusters found predominantly at MAR, HNL, and GYR (Actino 3, 4, and 5), (ii) the GYR DCM cluster (Actino 2), and (iii) the UPW cluster (Actino 1). One OTU identified as Actibacterium (see **Figure 2B**) was assigned to the same peak as the UPW Actinobacteria OTU. This OTU, although not detected at UPW, was present at low relative abundance at the other stations and could lead to a slight overestimation of the UPW OTU at these stations. Other assigned peaks included two joint *Prochlorococcus*/*Synechococcus* (Pro/Syn) peaks, and two SAR11 OTUs. The peaks assigned to SAR11 were also consistent with assignments done on bacterial communities from Antarctic waters (West et al., 2008). The Pro/Syn peaks are probably represented mainly by *Prochlorococcus* since from flow cytometry data (Grob et al., 2007), *Prochlorococcus* was from three to several orders of magnitude times more abundant than *Synechococcus* except from STA21 to the UPX where *Synechococcus* was more abundant than *Prochlorococcus*.

**FIGURE 5:**
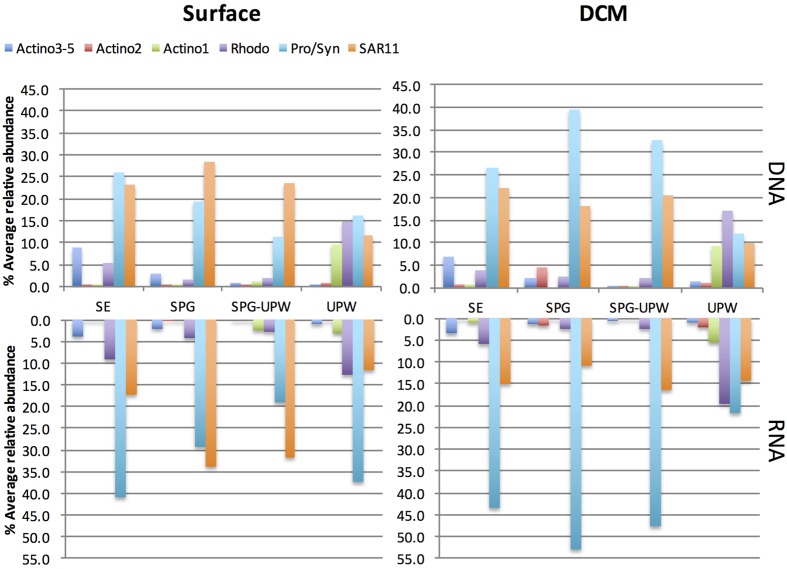
**Relative abundance of assigned SSCP peaks at surface or DCM depths in the RNA and DNA profiles averaged for the stations attributed to the four different water masses.** The *Prochlorococcus*/*Synechococcus* (Pro/Syn), SAR11 and Rhodobacteraceae (Rhodo) abundances are each composed of two peaks.

The relative presence (DNA) and activity (RNA) of the major SSCP OTUs that were assignable were averaged for each water mass at the surface and DCM depths (**Figure 5**). All peaks attributed to Actinobacteria showed a lower relative activity compared to their abundance in the DNA profiles. In surface waters at the western part of the transect, *Prochlorococcus* dominated the DNA and RNA profiles but its contribution decreased from the SE area to the SPG-UPW. Conversely, the SAR11 contribution to relative abundance and activity increased, reaching a maximum in the SPG. In the DCM, the average SAR11 contribution was fivefold lower than *Prochlorococcus* which contributed to over 50% of the RNA abundance. Two *Roseobacter* assigned OTUs belonging to the Rhodobacteraceae family (Rhodo in **Figure 5**) exhibited higher abundances at the phytoplankton-rich stations MAR and UPW in agreement with the clone library data. Their RNA/DNA ratios were for the majority >1. The three assigned Actinobacteria OTUs all showed lower contributions in the RNA profiles compared to that in the DNA profiles.

### Abiotic and Biotic Influences on Bacterioplankton Community Structure

The BIOSOPE transect passed through important environmental biotic and abiotic gradients including changes in temperature, nutrients, and phytoplankton biomass. Furthermore, phytoplankton diversity (represented essentially by cells <3 μm) changed significantly across the transect (Lepère et al., 2009; Shi et al., 2009, 2011a).

Ordination analysis was carried out for stations MAR to STB19 to explore the impact of biotic and abiotic variables on changes in bacterial diversity across the SPG. The eigenvalues of both the surface and DCM samples analyzed indicated significant species-environment correlations for the first (>0.97) and second axes (>0.96) with 36.1 and 30.6% of species variance explained by the first axes for surface and DCM samples, respectively. For the surface samples, the first axis was strongly correlated with temperature whereas the second axis was correlated with nutrients and phytoplankton abundance.

**FIGURE 6:**
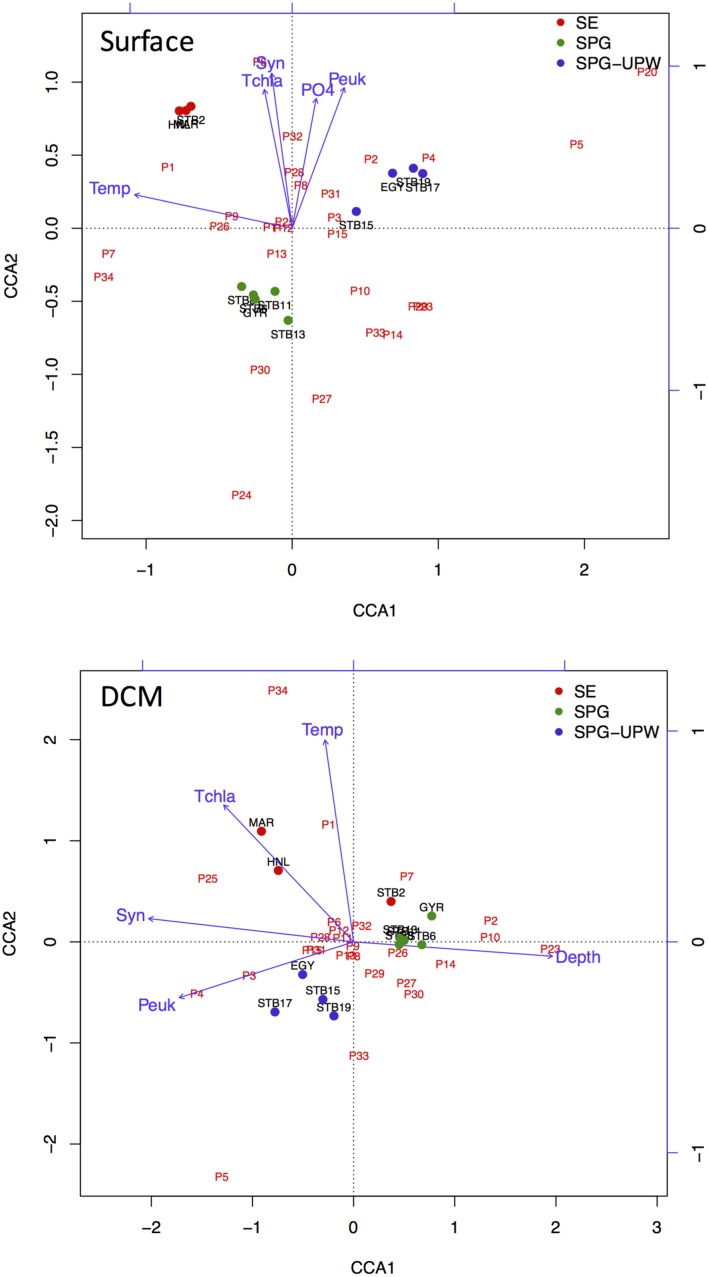
**CCA ordination triplots of Axes 1 and 2 for surface and DCM bacterial communities with respect to the environmental variables temperature (Temp), depth (Depth), nutrients phosphate (PO4), total chlorophyll *a* (Tchla), and picoplankton cell concentrations, *Synechococcus* (Syn) and picoeukaryote (Peuk).** Bacterial OTUs from SSCP peaks are indicated in red and stations from MAR to STB19 are color coded according to water mass.

For the DCM samples, the species variance was explained by opposing gradients of *Synechococcus* concentrations against depth on the first axis and by temperature on the second axis (**Figure 6**). To test the hypothesis that the variability of bacterial communities in the DCM may be more strongly influenced by phytoplankton-related variables, Mantel tests were performed between matrices of bacterial OTUs relative abundance (DNA SSCP data) against a set of environmental variables and against pigment data from the key phytoplankton groups. A summary of the results is presented in **Table 2.** The environmental variables were significantly correlated with the bacterial community structure for all depths (*r* = 0.262, *p* < 0.0001) but the correlation was stronger when only considering the surface depths (*r* > 0.5, *p* < 0.0001) and was non-significant for the DCM depths. Correlations with phytoplankton pigment concentrations were also significant with all depths, but higher correlations were obtained with the DCM depths (*r* = 0.892, *p* < 0.0001) than the surface depths (*r* = 0.412, *p* = 0.008).

**Table 2:** Mantel test results showing the significance of correlations between bacterial diversity (SSCP OTUs) and either environmental variables (env) or phytoplankton pigments (Pigments).

Bacterial diversity	Number of samples	Test	*r*	*p*
SSCP OTUs	21	Env	0.262	**<0.0001**
All depths		Pigments	0.653	**<0.0001**
SSCP OTUs	11	Env	0.514	**<0.0001**
Surface		Pigments	0.412	** 0.008**
SSCP OTUs	10	Env	0.183	0.205
DCM		Pigments	0.892	**<0.0001**


*Values in bold are significant at p < 0.005 (probability based on 999 permutations)*.

The relationship between specific environmental variables and the distribution of the Actinobacteria OTUs, was examined by Spearman correlations (**Table 3**). The Actinobacteria clusters found predominantly at MAR, HNL and GYR (Actino 3, 4, and 5) were significantly positively correlated with *Synechococcus* cell concentration followed by temperature and picoeukaryote cell concentration whereas the Actino 1 OTU was only positively correlated with phosphate and total chl*a* concentration.

**Table 3:** Spearman rank correlations between environmental variables and Actinobacteria OTUs dominant at MAR, HNL, and GYR (Actino 3–5) or at UPW (Actino 1).

	Syn	Pro	Peuk	Temp	Sal	PO4	NO3	TChla
Actino 1	n.s	–	n.s	n.s	n.s	0.64^∗^	n.s	0.73^∗^
Actino 3–5	0.89^∗∗∗^	n.s	0.68^∗^	0.80^∗∗^	-0.50^∗^	n.s	n.s	n.s


*Syn, Synechococcus cell abundance; Pro, Prochlorococcus cell abundance; Peuk, picoeukaryote cell abundance; Temp, temperature; Sal, salinity; PO4, phosphate concentration; NO3, nitrate concentration; Tchla, total chlorophyll a. Significance level*: ^∗∗∗^*p* < 0.0001, ^∗∗^*p* < 0.001, ^∗^*p <0.05, n.s, non-significant*.

## Discussion

### Microbial Diversity in the SPG

Conditions in the SPG are considered to be the most oligotrophic of the world’s oceans with total chl*a* concentrations <0.03 mg/m^-3^ (Ras et al., 2008) and with nitrate undetectable in surface waters (Raimbault et al., 2008). At the hyperoligotrophic GYR station, microbial communities were largely dominated (70% of all 16S rRNA gene sequences) by multiple OTUs of the SAR86 and SAR11 clades with lower contributions from groups affiliated to Actibacterium (Rhodobacteraceae), SAR116 and Actinobacteria. Not surprisingly, the GYR bacterial groups have been classified as typically oligotrophic from a single-cell genomics analysis of surface ocean bacterioplankton that used genomic signatures to infer trophic strategy (Swan et al., 2013). This study revealed that an oligotrophic lifestyle and photoheterotrophy is more widespread that was previously thought from sequenced genomes of cultured organisms. The dominance of SAR11 and SAR86 in the central gyre indicates that they have a selective advantage over the other oligotrophic groups. Such adaptations could include their small size, allowing more efficient absorption of nutrients, and their genome streamlining leading to resource specialization (Giovannoni et al., 2005b; Dupont et al., 2012; Grote et al., 2012). This specialization is particularly advantageous under stable environmental conditions such as those found within the central gyre and may explain why the microbial communities at both surface and DCM depths showed high similarity across the 1300 nautical miles distance of the SPG stations. The SAR11 and SAR86 clades can also enhance nutrient uptake by proteorhodopsin-conferred photo-heterotrophy (Béjà et al., 2000; Giovannoni et al., 2005a). Light enhanced uptake of organic molecules was shown for SAR11 in the North Atlantic gyre (Gómez-Pereira et al., 2013) and the majority of proteorhodopsin transcripts were attributed to SAR11/SAR11-like organisms in the North Pacific gyre (Shi et al., 2011b). Given the clear waters of this region (Morel et al., 2007) and the extreme nutrient limitation, photo-heterotrophy could provide a significant advantage to SAR11 and SAR86 in favoring competition for nutrients.

The DCM bacterial community was significantly different from the surface community showing a higher richness and phylogenetic diversity with increases in abundance of bacterial groups typically found at depth or associated with higher phytoplankton biomass including the Deferribacteres (SAR406), the Deltaproteobacteria (SAR324), Bacteroidetes, Actinobacteria and the OCS116 lineage. These groups were found in another SPG study using 16S rRNA tag sequences (Walsh et al., 2015) and also in the South Atlantic Gyre (Morris et al., 2012) and the North Atlantic Gyre (Treusch et al., 2009). Relative abundances of *Prochlorococcus* also increased with depth in agreement with flow cytometry cell abundance data (Grob et al., 2007).

### Changes in Bacterial Diversity and Activity across the South Pacific Basin

Striking patterns of horizontal and vertical spatial variability across the eastern South Pacific basin were observed for all the major bacterial groups including SAR11, SAR86, Actinobacteria, *Rhodobacteraceae* and Bacteroidetes as observed for the South Atlantic basin (Morris et al., 2012). Not only do we show spatial differences in the SAR11 subclades as observed previously (Morris et al., 2012), but we also present evidence of similar differential distributions within the SAR86 clade and the Actinobacteria. For the SAR11 subclades, similarities between the South Atlantic and South Pacific basins were observed: Ia was the most abundant subclade found in the clone libraries and was distributed across the transect, accounting for the highest proportion of total SAR11 sequences in the upwelling, as also observed in the South Atlantic (Morris et al., 2012). In contrast, at the GYR DCM, Ia showed low relative abundances and was instead replaced by subclades Ib and II. Subclade Ib also accounted for a higher relative abundance in the warmer surface waters of the gyre station in agreement with its observed biogeography (Brown et al., 2012). In contrast to the Sargasso Sea, where subclade Ib was replaced with Ia at the onset of summer stratification, in the highly stratified waters of the gyre Ia and Ib were present at roughly equal contributions in our clone libraries.

Of the assigned bacterial groups in the fingerprint profiles, SAR11 and *Prochlorococcus* showed the highest relative abundance and activity in all water masses except for the upwelling. In surface waters, SAR11 increased in abundance and potential activity toward the SPG to the contrary of *Prochlorococcus* which showed its highest abundance and potential activity in the sub-equatorial area (SE) where cell abundances reached a maximum at station STB2 (Grob et al., 2007). High activity of *Prochlorococcus* and SAR11 in surface waters was also inferred from a metatranscriptomic study in the North Pacific gyre where these groups dominated 16S rRNA reads from cDNA libraries and also protein-coding transcripts (Shi et al., 2011b). The lower relative activity of SAR11 at DCM depths that we observed is also in agreement with this previous study that indicated a lower transcriptional activity of SAR11 at DCM depths (Shi et al., 2011b).

The SAR86 clade showed a different distribution pattern to that observed in the South Atlantic – higher SAR86 relative abundances were in the Benguela upwelling whereas in this study, slightly higher relative abundances were observed in surface GYR waters. The use of longer clone library sequences combined with discrimination of OTUs at a lower taxonomic level (99% similarity) allowed us to show that the SAR86 subclade II was only detected in the colder waters of the upwelling whereas subclade I was distributed in the warmer waters from MAR to GYR in agreement with analyses of the GOS metagenome dataset (Rusch et al., 2007; Dupont et al., 2012). Although, based on a low number of clones, there was evidence for distinguishable SAR86 OTUs between the SE area stations MAR and HNL and the SPG station GYR. A similar spatial pattern was also observed for Actinobacteria OTUs (Actino 4 and Actino 5; **Figure 2**) and *Prochlorococcus* (West et al., 2011) and may suggest that there is ecotype partitioning along environmental gradients between the SE and SPG water masses.

Actinobacteria showed the highest relative abundance (around 20% of all bacterial sequences) in surface waters at the phytoplankton rich stations MAR and UPW. In addition, they were also dominant members of bacterioplankton communities in phytoplankton rich mesoscale cyclonic eddies (Zhang et al., 2011) and in a diatom enriched eddy (Nelson et al., 2013). Interestingly, the Actinobacteria OTUs that were most abundant at MAR and HNL fall into a newly defined cluster of ultra-small marine (∼0.3 μm) Actinobacteria that possesses a rhodopsin gene tuned for green light that would be particularly adapted for light harvesting in more productive waters (Ghai et al., 2013). Although, in the Actinobacteria tree presented here (**Figure 4**), the MAR-HNL-GYR cluster affiliated with this new group and was separated from the others (**Figure 4**), all of these clusters *matched* with the specific sub-class ‘*Candidatus* Actinomarinidae’ probe (Ghai et al., 2013) whilst the branching of the MAR-HNL-GYR cluster within the OCS155 clade had low support (65%). Our data lend new insights into the distribution of marine Actinobacteria showing that the UPW OCS155/OM1 Actinobacteria OTU and the MAR-HNL-GYR ‘*Ca*. Actinomarinidae’ OTU display opposite spatial distributions that decrease in relative abundance toward the GYR station (**Figures 3B and 5**). A distinct OTU related to the OCS155/OM1 group was only present at the GYR DCM and is probably closely related to the Actinobacteria clones recovered from the DCM in the South Atlantic Gyre (Morris et al., 2012). As may be expected in more productive areas, the highest relative activities of the three assigned Actinobacteria OTUs was observed at the MAR and UPW stations.

### Influence of Abiotic and Biotic Variables on Microbial Diversity

In surface waters, the temperature/salinity gradient was the most significant variable explaining the variability of the bacterial communities as reflected in the CCA ordination (**Figure 6**) and supported by the ANOSIM test showing significant differences between bacterial communities in different water masses. The partitioning of microbial communities according to water mass was shown in the Atlantic Ocean (Agogué et al., 2011) and in the Arctic (Hamilton et al., 2008; Galand et al., 2010). The separation of these communities can be due to dispersal limitation due to differential water densities acting as a physical barrier, but may also be due to the selection of communities adapted to the *in situ* environmental conditions. An increasing number of studies point to temperature and latitude as major drivers of marine microbial community structure rather than dispersal limitation (Fuhrman et al., 2008; Swan et al., 2013) and they were also the main variables explaining the distribution of SAR11 phylotypes on a global scale (Brown et al., 2012). The SPG was highly stratified (Claustre et al., 2008) and this was reflected in the distinct bacterial communities at the surface and DCM depths. At the DCM depths there was evidence of bacterial community structuring by phytoplankton conferred variables and depth as indicated by the CCA ordinations and the Mantel tests which showed a higher correlation between the species composition matrix and the phytoplankton conferred variables compared to all environmental variables combined. These results are also in agreement with Walsh et al. (2015) who showed significant correlations between bacterial community composition with depth and sea surface chlorophyll a concentrations for the SPG DCM. Whereas the above analyses consider the whole bacterial community, correlations at a finer taxonomic level, at the level of an OTU or ecotype can reveal the influence of different environmental variables. The *Actinobacteria* OTUs detected from stations MAR-GYR were highly correlated with temperature and *Synechococcus* cell concentration. This latter relationship has already been observed for this widely distributed clade of Actinobacteria (Ghai et al., 2013) and may indicate a specific interaction between these two groups.

## Conclusion

In this study we combined a 16S rRNA clone library and RNA–DNA fingerprinting approach to gain insights into the phylogenetic diversity and potential activity of the bacterial communities across the South Pacific Ocean crossing the unique hyperoligotrophic SPG. Sampling of surface and DCM depths allowed us to show that while the bacterial communities in each depth layer across the central gyre stations showed a high similarity across >1300 nautical miles, the surface and DCM communities at a given station were significantly different. Distinct distributions of OTUs of the major clades SAR11, SAR86, Actinobacteria across the transect were similar to that observed in the South Atlantic Ocean (Morris et al., 2012) with temperature the most important variable influencing the structuring of the microbial communities. The SPG was characterized by a dominance of typical oligotrophic-adapted bacteria including the heterotrophs SAR11 and SAR86 and the phototroph *Prochlorococcus*. Whereas the potential activity of SAR11 decreased with depth, the converse was observed for *Prochlorococcus.* We speculate that if these groups converge on a photoheterotrophic lifestyle under oligotrophic conditions to enhance uptake of organic molecules, the PR-conferred mixotrophy of SAR11 may be less efficient under low light conditions at DCM depths.

## Author Contributions

NW performed the experiments, did the data analysis and wrote the paper. CL contributed statistical analysis and to the writing of the paper. C-LM did the DNA extractions and PC collected the samples. DS and PL contributed to the writing of the paper and the PICOFUNPAC project.

## Conflict of Interest Statement

The authors declare that the research was conducted in the absence of any commercial or financial relationships that could be construed as a potential conflict of interest.

## References

[B1] Acinas S. G. Klepac-Ceraj V. Hunt D. E. Pharino C. Ceraj I. Distel D. L. (2004). Fine-scale phylogenetic architecture of a complex bacterial community. *Nature* 430 551–554. 10.1038/nature02649 15282603

[B2] Agogué H. Lamy D. Neal P. R. Sogin M. L. Herndl G. J. (2011). Water mass-specificity of bacterial communities in the North Atlantic revealed by massively parallel sequencing. *Mol. Ecol.* 20 258–274. 10.1111/j.1365-294X.2010.04932.x 21143328 PMC3057482

[B3] Altekar G. Dwarkadas S. Huelsenbeck J. P. Ronquist F. (2004). Parallel metropolis coupled markov chain monte carlo for *Bayesian phylogenetic* inference. *Bioinformatics* 20 407–415. 10.1093/bioinformatics/btg427 14960467

[B4] Ashelford K. E. Chuzhanova N. A. Fry J. C. Jones A. J. Weightman A. J. (2005). At least 1 in 20 16S rRNA sequence records currently held in public repositories is estimated to contain substantial anomalies. *Appl. Environ. Microbiol.* 71 7724–7736. 10.1128/AEM.71.12.7724-7736.2005 16332745 PMC1317345

[B5] Ashelford K. E. Chuzhanova N. A. Fry J. C. Jones A. J. Weightman A. J. (2006). New screening software shows that most recent large 16S rRNA gene clone libraries contain chimeras. *Appl. Environ. Microbiol.* 72 5734–5741. 10.1128/AEM.00556-06 16957188 PMC1563593

[B6] Béjà O. Aravind L. Koonin E. V. Suzuki M. T. Hadd A. Nguyen L. P. (2000). Bacterial rhodopsin: evidence for a new type of phototrophy in the sea. *Science* 289 1902–1906. 10.1126/science.289.5486.1902 10988064

[B7] Blazewicz S. J. Barnard R. L. Daly R. A. Firestone M. K. (2013). Evaluating rRNA as an indicator of microbial activity in environmental communities: limitations and uses. *ISME J.* 7 2061–2068. 10.1038/ismej.2013.102 23823491 PMC3806256

[B8] Brown M. Schwalbach M. Hewson I. Fuhrman J. (2005). Coupling 16S-ITS rDNA clone libraries and automated ribosomal intergenic spacer analysis to show marine microbial diversity: development and application to a time series. *Environ. Microbiol.* 7 1466–1479. 10.1111/j.1462-2920.2005.00835.x 16104869

[B9] Brown M. V. Fuhrman J. A. (2005). Marine bacterial microdiversity as revealed by internal transcribed spacer analysis. *Aquat. Microbiol. Ecol.* 41 15–23. 10.3354/ame041015

[B10] Brown M. V. Lauro F. M. DeMaere M. Z. Muir L. Wilkins D. Thomas T. (2012). Global biogeography of SAR11 marine bacteria. *Mol. Syst. Biol.* 8:595 10.1038/msb.2012.28 PMC3421443 22806143

[B11] Carlson C. A. Morris R. Parsons R. Treusch A. H. Giovannoni S. J. Vergin K. (2009). Seasonal dynamics of SAR11 populations in the euphotic and mesopelagic zones of the northwestern Sargasso Sea. *ISME J.* 3 283–295. 10.1038/ismej.2008.117 19052630

[B12] Claustre H. Sciandra A. Vaulot D. (2008). Introduction to the special section bio-optical and biogeochemical conditions in the South East Pacific in late 2004: the BIOSOPE program. *Biogeosciences* 5 679–691. 10.5194/bg-5-679-2008

[B13] Delbès C. Godon J.-J. Moletta R. (1998). 16S rDNA sequence diversity of a culture-accessible part of an anaerobic digestor bacterial community. *Anaerobe* 4 267–275. 10.1006/anae.1998.0176 16887652

[B14] Delbès C. Moletta R. Godon J.-J. (2000). Monitoring of activity dynamics of an anaerobic digester bacterial community using 16S rRNA polymerase chain reaction–single-strand conformation polymorphism analysis. *Environ. Microbiol.* 2 506–515. 10.1046/j.1462-2920.2000.00132.x 11233159

[B15] Dupont C. L. Rusch D. B. Yooseph S. Lombardo M.-J. Richter R. A. Valas R. (2012). Genomic insights to SAR86, an abundant and uncultivated marine bacterial lineage. *ISME J.* 6 1186–1199. 10.1038/ismej.2011.189 22170421 PMC3358033

[B16] Edgar R. C. (2010). Search and clustering orders of magnitude faster than BLAST. *Bioinformatics* 26 2460–2461. 10.1093/bioinformatics/btq461 20709691

[B17] Edgar R. C. Haas B. J. Clemente J. C. Quince C. Knight R. (2011). UCHIME improves sensitivity and speed of chimera detection. *Bioinform. Oxf. Engl.* 27 2194–2200. 10.1093/bioinformatics/btr381 PMC3150044 21700674

[B18] Eiler A. Hayakawa D. H. Church M. J. Karl D. M. Rappé M. S. (2009). Dynamics of the SAR11 bacterioplankton lineage in relation to environmental conditions in the oligotrophic North Pacific subtropical gyre. *Environ. Microbiol.* 11 2291–2300. 10.1111/j.1462-2920.2009.01954.x 19490029

[B19] Fuhrman J. A. Steele J. A. (2008). Community structure of marine bacterioplankton: patterns, networks, and relationships to function. *Aquat. Microbiol. Ecol.* 53 69–81. 10.3354/ame01222

[B20] Fuhrman J. A. Steele J. A. Hewson I. Schwalbach M. S. Brown M. V. Green J. L. (2008). A latitudinal diversity gradient in planktonic marine bacteria. *Proc. Natl. Acad. Sci. U.S.A.* 105 7774–7778. 10.1073/pnas.0803070105 18509059 PMC2409396

[B21] Galand P. E. Potvin M. Casamayor E. O. Lovejoy C. (2010). Hydrography shapes bacterial biogeography of the deep Arctic Ocean. *ISME J.* 4 564–576. 10.1038/ismej.2009.134 20010630

[B22] Ghai R. Mizuno C. M. Picazo A. Camacho A. Rodriguez-Valera F. (2013). Metagenomics uncovers a new group of low GC and ultra-small marine Actinobacteria. *Sci. Rep.* 3:2471 10.1038/srep02471 PMC3747508 23959135

[B23] Giovannoni S. J. Bibbs L. Cho J. C. Stapels M. D. Desiderio R. Vergin K. L. (2005a). Proteorhodopsin in the ubiquitous marine bacterium SAR11. *Nature* 438 82–85. 10.1038/nature04032 16267553

[B24] Giovannoni S. J. Tripp H. J. Givan S. Podar M. Vergin K. L. Baptista D. (2005b). Genome streamlining in a cosmopolitan oceanic bacterium. *Science* 309 1242–1245. 10.1126/science.1114057 16109880

[B25] Giovannoni S. J. Vergin K. L. (2012). Seasonality in ocean microbial communities. *Science* 335 671–676. 10.1126/science.1198078 22323811

[B26] Gómez-Pereira P. R. Hartmann M. Grob C. Tarran G. A. Martin A. P. Fuchs B. M. (2013). Comparable light stimulation of organic nutrient uptake by SAR11 and Prochlorococcus in the North Atlantic subtropical gyre. *ISME J.* 7 603–614. 10.1038/ismej.2012.126 23096403 PMC3580278

[B27] Grob C. Ulloa O. Claustre H. Huot Y. Alarcon G. Marie D. (2007). Contribution of picoplankton to the total particulate organic carbon concentration in the eastern South Pacific. *Biogeosciences* 4 837–852. 10.5194/bg-4-837-2007

[B28] Grote J. Thrash J. C. Huggett M. J. Landry Z. C. Carini P. Giovannoni S. J. (2012). Streamlining and core genome conservation among highly divergent members of the SAR11 clade. *mBio* 3:e252 10.1128/mBio.00252-12 PMC3448164 22991429

[B29] Hamilton A. K. Lovejoy C. Galand P. E. Ingram R. G. (2008). Water masses and biogeography of picoeukaryote assemblages in a cold hydrographically complex system. *Limnol. Oceanogr.* 53 922–935. 10.4319/lo.2008.53.3.0922

[B30] Jensen P. R. Lauro F. M. (2008). An assessment of actinobacterial diversity in the marine environment. *Antonie Van Leeuwenhoek* 94 51–62. 10.1007/s10482-008-9239-x 18500568 PMC3375478

[B31] Karl D. M. Christian J. R. Dore J. E. Hebel D. V. Letelier R. M. Tupas L. M. (1996). Seasonal and interannual variability in primary production and particle flux at Station ALOHA. *Deep Sea Res. Part II Top. Stud. Oceanogr.* 43 539–568. 10.1016/0967-0645(96)00002-1

[B32] Lee D. H. Zo Y. G. Kim S. J. (1996). Nonradioactive method to study genetic profiles of natural bacterial communities by PCR-single-strand-conformation polymorphism. *Appl. Environ. Microbiol.* 62 3112–3120. 8795197 10.1128/aem.62.9.3112-3120.1996 PMC168103

[B33] Lepère C. Vaulot D. Scanlan D. J. (2009). Photosynthetic picoeukaryote community structure in the South East Pacific Ocean encompassing the most oligotrophic waters on Earth. *Environ. Microbiol.* 11 3105–3117. 10.1111/j.1462-2920.2009.02015.x 19674042

[B34] Lozupone C. Knight R. (2005). UniFrac: a new phylogenetic method for comparing microbial communities. *Appl. Environ. Microbiol.* 71 8228–8235. 10.1128/AEM.71.12.8228-8235.2005 16332807 PMC1317376

[B35] Malmstrom R. R. Straza T. R. A. Cottrell M. T. Kirchman D. L. (2007). Diversity, abundance, and biomass production of bacterial groups in the western Arctic Ocean. *Aquat. Microbiol. Ecol.* 47 45–55. 10.3354/ame047045

[B36] Manes C.-L. West N. J. Rapenne S. Lebaron P. (2010). Dynamic bacterial communities on reverse-osmosis membranes in a full-scale desalination plant. *Biofouling* 27 47–58. 10.1080/08927014.2010.536980 21108068

[B37] Mantel N. (1967). The detection of disease clustering and a generalized regression approach. *Cancer Res.* 27 209–220. 6018555

[B38] Morel A. Gentili B. Claustre H. Babin M. Bricaud A. Ras J. (2007). Optical properties of the “clearest” natural waters. *Limnol. Oceanogr.* 52 217–229. 10.1364/AO.20.000177

[B39] Morris R. M. Frazar C. D. Carlson C. A. (2012). Basin-scale patterns in the abundance of SAR11 subclades, marine *Actinobacteria* (OM1), members of the Roseobacter clade and OCS116 in the South Atlantic. *Environ. Microbiol.* 14 1133–1144. 10.1111/j.1462-2920.2011.02694.x 22225975

[B40] Morris R. M. Nunn B. L. Frazar C. Goodlett D. R. Ting Y. S. Rocap G. (2010). Comparative metaproteomics reveals ocean-scale shifts in microbial nutrient utilization and energy transduction. *ISME J.* 4 673–685. 10.1038/ismej.2010.4 20164862

[B41] Morris R. M. Rappe M. S. Connon S. A. Vergin K. L. Siebold W. A. Carlson C. A. (2002). SAR11 clade dominates ocean surface bacterioplankton communities. *Nature* 420 806–810. 10.1038/nature01240 12490947

[B42] Morris R. M. Vergin K. L. Cho J. C. Rappe M. S. Carlson C. A. Giovannoni S. J. (2005). Temporal and spatial response of bacterioplankton lineages to annual convective overturn at the Bermuda Atlantic Time-series Study site. *Limnol. Oceanogr.* 50 1687–1696. 10.4319/lo.2005.50.5.1687

[B43] Needham D. M. Chow C.-E. T. Cram J. A. Sachdeva R. Parada A. Fuhrman J. A. (2013). Short-term observations of marine bacterial and viral communities: patterns, connections and resilience. *ISME J.* 7 1274–1285. 10.1038/ismej.2013.19 23446831 PMC3695287

[B44] Nelson C. E. Carlson C. A. Ewart C. S. Halewood E. R. (2013). Community differentiation and population enrichment of Sargasso Sea bacterioplankton in the euphotic zone of a mesoscale mode-water eddy. *Environ. Microbiol.* 16 871–887. 10.1111/1462-2920.12241 24589288

[B45] Polovina J. J. Howell E. A. Abecassis M. (2008). Ocean’s least productive waters are expanding. *Geophys. Res. Lett.* 35:L03618 10.1029/2007GL031745

[B46] Raimbault P. Garcia N. Cerutti F. (2008). Distribution of inorganic and organic nutrients in the South Pacific Ocean - evidence for long-term accumulation of organic matter in nitrogen-depleted waters. *Biogeosciences* 5 281–298. 10.5194/bg-5-281-2008

[B47] Ras J. Claustre H. Uitz J. (2008). Spatial variability of phytoplankton pigment distributions in the Subtropical South Pacific Ocean: comparison between in situ and predicted data. *Biogeosciences* 5 353–369. 10.5194/bg-5-353-2008

[B48] R Core Team (2015). *R: A Language and Environment for Statistical Computing.* Vienna: R Foundation for Statistical Computing. Available at: https://www.R-project.org/

[B49] Rusch D. B. Halpern A. L. Sutton G. Heidelberg K. B. Williamson S. Yooseph S. (2007). The sorcerer II Global Ocean Sampling expedition: Northwest Atlantic through Eastern Tropical Pacific. *PLoS Biol.* 5:e77 10.1371/journal.pbio.0050077 PMC1821060 17355176

[B50] Salter I. Galand P. E. Fagervold S. K. Lebaron P. Obernosterer I. Oliver M. J. (2015). Seasonal dynamics of active SAR11 ecotypes in the oligotrophic Northwest Mediterranean Sea. *ISME J.* 9 347–360. 10.1038/ismej.2014.129 25238399 PMC4303628

[B51] Schloss P. D. Westcott S. L. Ryabin T. Hall J. R. Hartmann M. Hollister E. B. (2009). Introducing mothur: open-source, platform-independent, community-supported software for describing and comparing microbial communities. *Appl. Environ. Microbiol.* 75 7537–7541. 10.1128/AEM.01541-09 19801464 PMC2786419

[B52] Shi X. L. Lepère C. Scanlan D. J. Vaulot D. (2011a). Plastid 16S rRNA Gene diversity among eukaryotic picophytoplankton sorted by flow cytometry from the South Pacific Ocean. *PLoS ONE* 6:e18979 10.1371/journal.pone.0018979 PMC3084246 21552558

[B53] Shi X. L. Marie D. Jardillier L. Scanlan D. J. Vaulot D. (2009). Groups without cultured representatives dominate eukaryotic picophytoplankton in the oligotrophic South East Pacific Ocean. *PLoS ONE* 4:e7657 10.1371/journal.pone.0007657 PMC2764088 19893617

[B54] Shi Y. Tyson G. W. Eppley J. M. DeLong E. F. (2011b). Integrated metatranscriptomic and metagenomic analyses of stratified microbial assemblages in the open ocean. *ISME J.* 5 999–1013. 10.1038/ismej.2010.189 21151004 PMC3131857

[B55] Suzuki M. T. Beja O. Taylor L. T. DeLong E. F. (2001). Phylogenetic analysis of ribosomal RNA operons from uncultivated coastal marine bacterioplankton. *Environ. Microbiol.* 3 323–331. 10.1046/j.1462-2920.2001.00198.x 11422319

[B56] Suzuki M. T. Preston C. M. Beja O. de la Torre J. R. Steward G. F. DeLong E. F. (2004). Phylogenetic screening of ribosomal RNA gene-containing clones in bacterial artificial chromosome (BAC) libraries from different depths in Monterey Bay. *Microbiol. Ecol.* 48 473–488. 10.1007/s00248-004-0213-5 15696381

[B57] Swan B. K. Tupper B. Sczyrba A. Lauro F. M. Martinez-Garcia M. González J. M. (2013). Prevalent genome streamlining and latitudinal divergence of planktonic bacteria in the surface ocean. *Proc. Natl. Acad. Sci. U.S.A.* 110 11463–11468. 10.1073/pnas.1304246110 23801761 PMC3710821

[B58] Thompson J. R. Marcelino L. A. Polz M. F. (2002). Heteroduplexes in mixed-template amplifications: formation, consequence and elimination by ‘reconditioning PCR’. *Nucleic Acids Res.* 30 2083–2088. 10.1093/nar/30.9.2083 11972349 PMC113844

[B59] Treusch A. H. Vergin K. L. Finlay L. A. Donatz M. G. Burton R. M. Carlson C. A. (2009). Seasonality and vertical structure of microbial communities in an ocean gyre. *ISME J.* 3 1148–1163. 10.1038/ismej.2009.60 19494846

[B60] Van Wambeke F. Bonnet S. Moutin T. Raimbault P. Alarcon G. Guieu C. (2008a). Factors limiting heterotrophic bacterial production in the southern Pacific Ocean. *Biogeosciences* 5 833–845. 10.5194/bg-5-833-2008

[B61] Van Wambeke F. Obernosterer I. Moutin T. Duhamel S. Ulloa O. Claustre H. (2008b). Heterotrophic bacterial production in the eastern South Pacific: longitudinal trends and coupling with primary production. *Biogeosciences* 5 157–169. 10.5194/bg-5-157-2008

[B62] Vergin K. L. Beszteri B. Monier A. Cameron Thrash J. Temperton B. Treusch A. H. (2013). High-resolution SAR11 ecotype dynamics at the Bermuda Atlantic Time-series Study site by phylogenetic placement of pyrosequences. *ISME J.* 7 1322–1332. 10.1038/ismej.2013.32 23466704 PMC3695298

[B63] Vergin K. L. Urbach E. Stein J. L. DeLong E. F. Lanoil B. D. Giovannoni S. J. (1998). Screening of a fosmid library of marine environmental genomic DNA fragments reveals four clones related to members of the order Planctomycetales. *Appl. Environ. Microbiol.* 64 3075–3078. 9687477 10.1128/aem.64.8.3075-3078.1998 PMC106819

[B64] Walsh E. A. Smith D. C. Sogin M. L. DHondt S. (2015). FEATURE ARTICLE Bacterial and archaeal biogeography of the deep chlorophyll maximum in the South Pacific Gyre. *Aquat. Microbiol. Ecol.* 75 1–13. 10.3354/ame01746

[B65] West N. J. Lebaron P. Strutton P. G. Suzuki M. T. (2011). A novel clade of Prochlorococcus found in high nutrient low chlorophyll waters in the South and Equatorial Pacific Ocean. *ISME J.* 5 933–944. 10.1038/ismej.2010.186 21124492 PMC3131852

[B66] West N. J. Obernosterer I. Zemb O. Lebaron P. (2008). Major differences of bacterial diversity and activity inside and outside of a natural iron-fertilized phytoplankton bloom in the Southern Ocean. *Environ. Microbiol.* 10 738–756. 10.1111/j.1462-2920.2007.01497.x 18237307

[B67] Yin Q. Fu B. Li B. Shi X. Inagaki F. Zhang X.-H. (2013). Spatial variations in microbial community composition in surface seawater from the ultra-oligotrophic center to rim of the South Pacific Gyre. *PLoS ONE* 8:e55148 10.1371/journal.pone.0055148 PMC3566182 23405118

[B68] Zhang Y. Jiao N. Sun Z. Hu A. Zheng Q. (2011). Phylogenetic diversity of bacterial communities in South China Sea mesoscale cyclonic eddy perturbations. *Res. Microbiol.* 162 320–329. 10.1016/j.resmic.2010.12.006 21187147

