# Supplementary material for: Distinct Spatial Patterns of SAR11, SAR86, and Actinobacteria Diversity along a Transect in the Ultra-oligotrophic South Pacific Ocean
Source: Front Microbiol. 2016 Mar 8;7:234. doi: 10.3389/fmicb.2016.00234 (PMC4781884; doi:10.3389/fmicb.2016.00234)

**Supplementary Information**

Fig. S1. Rarefaction curves for the surface and DCM 16S rRNA gene clone libraries. Sample depths are indicated after the station name.


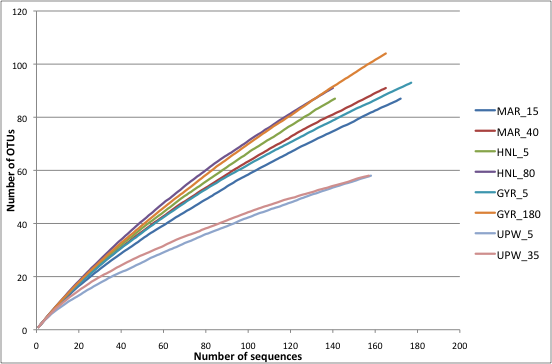


Fig S2. Upper panel: Similarity of surface (S) and DCM (D) bacterial communities (SSCP profiles) from the BIOSOPE transect. Lower panel: nMDS plot of the similarity of surface bacterial communities across the BIOSOPE transect with the trajectory of the transect overlaid.


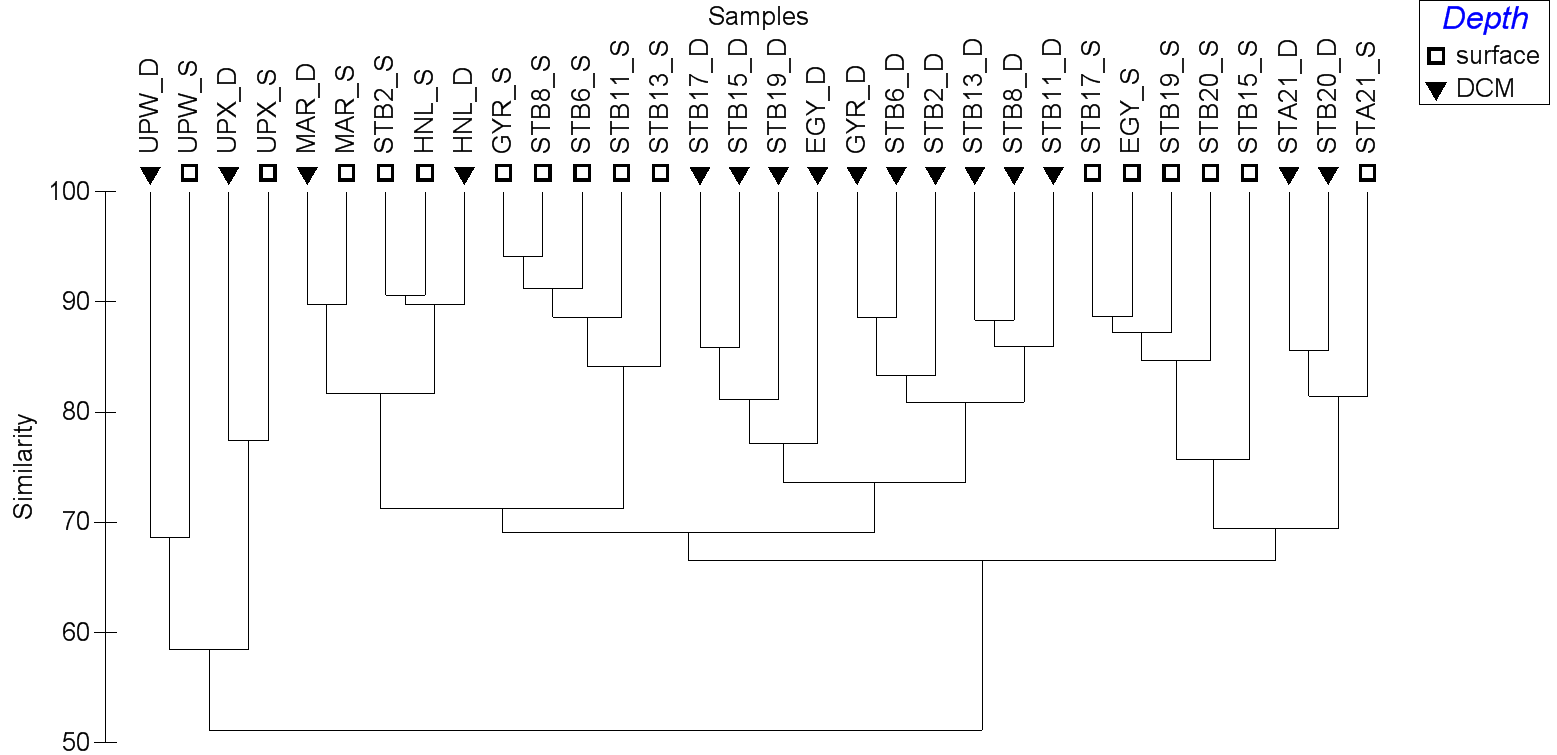


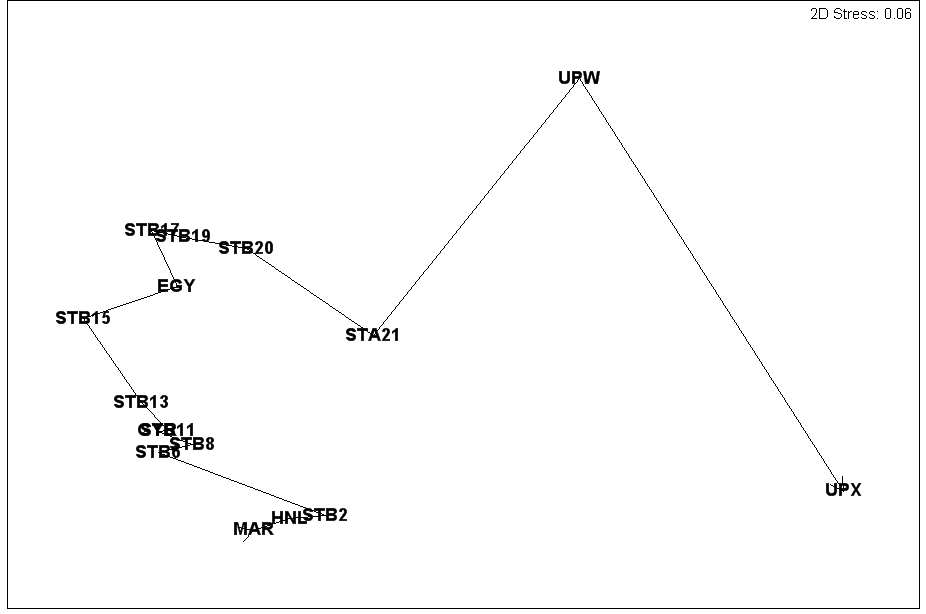

Supplement: Supplementary file 1 [file Data_Sheet_1.DOC]
